# Promising prospective effects of *Withania somnifera* on broiler performance and carcass characteristics: A comprehensive review

**DOI:** 10.3389/fvets.2022.918961

**Published:** 2022-09-02

**Authors:** Heba M. Salem, Mohamed T. El-Saadony, Taia A. Abd El-Mageed, Soliman M. Soliman, Asmaa F. Khafaga, Ahmed M. Saad, Ayman A. Swelum, Sameh A. Korma, Clara Mariana Gonçalves Lima, Samy Selim, Ahmad O. Babalghith, Mohamed E. Abd El-Hack, Fatima A. Omer, Synan F. AbuQamar, Khaled A. El-Tarabily, Carlos Adam Conte-Junior

**Affiliations:** ^1^Department of Poultry Diseases, Faculty of Veterinary Medicine, Cairo University, Giza, Egypt; ^2^Department of Agricultural Microbiology, Faculty of Agriculture, Zagazig University, Zagazig, Egypt; ^3^Soil and Water Department, Faculty of Agriculture, Fayoum University, Fayoum, Egypt; ^4^Department of Medicine and Infectious Diseases, Faculty of Veterinary Medicine, Cairo University, Giza, Egypt; ^5^Department of Pathology, Faculty of Veterinary Medicine, Alexandria University, Alexandria, Egypt; ^6^Biochemistry Department, Faculty of Agriculture, Zagazig University, Zagazig, Egypt; ^7^Department of Animal Production, College of Food and Agriculture Sciences, King Saud University, Riyadh, Saudi Arabia; ^8^Department of Theriogenology, Faculty of Veterinary Medicine, Zagazig University, Zagazig, Egypt; ^9^Department of Food Science, Faculty of Agriculture, Zagazig University, Zagazig, Egypt; ^10^Department of Food Science, Federal University of Lavras, Lavras, Minas Gerais, Brazil; ^11^Department of Clinical Laboratory Sciences, College of Applied Medical Sciences, Jouf University, Sakaka, Saudi Arabia; ^12^Medical Genetics Department, College of Medicine, Umm Al-Qura University, Makkah, Saudi Arabia; ^13^Poultry Department, Faculty of Agriculture, Zagazig University, Zagazig, Egypt; ^14^Department of Biology, College of Science, United Arab Emirates University, Al-Ain, United Arab Emirates; ^15^Khalifa Center for Genetic Engineering and Biotechnology, United Arab Emirates University, Al-Ain, United Arab Emirates; ^16^Harry Butler Institute, Murdoch University, Murdoch, WA, Australia; ^17^Center for Food Analysis (NAL), Technological Development Support Laboratory (LADETEC), Federal University of Rio de Janeiro (UFRJ), Cidade Universitária, Rio de Janeiro, Brazil

**Keywords:** antioxidant, birds' productivity, herbal extract, poultry, *Withania somnifera*

## Abstract

Poultry production contributes markedly to bridging the global food gap. Many nations have limited the use of antibiotics as growth promoters due to increasing bacterial antibiotic tolerance/resistance, as well as the presence of antibiotic residues in edible tissues of the birds. Consequently, the world is turning to use natural alternatives to improve birds' productivity and immunity. *Withania somnifera*, commonly known as ashwagandha or winter cherry, is abundant in many countries of the world and is considered a potent medicinal herb because of its distinct chemical, medicinal, biological, and physiological properties. This plant exhibits antioxidant, cardioprotective, immunomodulatory, anti-aging, neuroprotective, antidiabetic, antimicrobial, antistress, antitumor, hepatoprotective, and growth-promoting activities. In poultry, dietary inclusion of *W. somnifera* revealed promising results in improving feed intake, body weight gain, feed efficiency, and feed conversion ratio, as well as reducing mortality, increasing livability, increasing disease resistance, reducing stress impacts, and maintaining health of the birds. This review sheds light on the distribution, chemical structure, and biological effects of *W. somnifera* and its impacts on poultry productivity, livability, carcass characteristics, meat quality, blood parameters, immune response, and economic efficiency.

## Introduction

Global food production predominantly depends on animal protein. In many nations, the poultry business has grown in importance as a resource of high-quality eggs and meat to help balance the human food (1). The nutritional economic demands of various countries for a poultry-based diet have forced the intensive production of poultry (2). Furthermore, backyard poultry production is gradually evolving into economically organized flocks and is considered a competitive and rapidly growing section of animal-farming business (3). However, the global poultry industry is facing numerous challenges of sufficiency, safer products without any chemical and antimicrobial residues, and environmentally sustainable production (4). These conditions have led to the discovery and abundant use of various natural and safe feed additives that can be included in the poultry ration to improve productivity through a variety of mechanisms, such as boosting growth rate, enhancing feed conversion efficiency, decreasing pathogen propagation, increasing livability, and decreasing mortality in the poultry industry (4).

The feed additive should be safe, economic, biodegradable, free from environmental hazards, and non-toxic, as well as overcoming drug resistance problems and improving productivity (5). Thus, an eco-friendly substitution of antibacterial growth promoters (AGPs) with a natural growth promoter in the avian ration has recently acquired considerable attention (6, 7) to improve productivity and fight infections (8). Many natural growth promoters (NGPs) such as herbal extracts (9–11), probiotics (12–15), prebiotics (16, 17), phytogenic compounds (18–22), bioactive peptides (23–25), essential oils (26–28), organic acids (29), plants and their active constituents (30–35), and green-synthesized nanoparticles (36–41) are recognized as potential and safe alternatives to AGPs (42). The use of medicinal plants as feed additives to boost development and health is becoming increasingly common around the world (43, 44) owing to the unique properties of these plants, including low cost, low toxicity risk, and minimum human health and environmental hazards (45).

Traditional medicinal herbs are common therapeutics and more potent in combating the negative impacts of thermal stress on broiler productivity (44). The predominant mechanism by which medicinal herbs act in avian rations is to improve the metabolism by combating stress and regulating hormones (46). Numerous field studies on medicinal herbs from all over the world have revealed promising outputs in improving weight gain (WG) and feed efficiency, reducing mortality, elevating livability, and maintaining health among different avian species (47–49).

One of these medicinal herbs is *Withania somnifera* L. Dunal, commonly known as “ashwagandha” or “winter cherry” (50). *W. somnifera* is a subtropical plant of 30–150 cm height that belongs to the family Solanaceae and grows naturally in wide areas of Africa, the East Mediterranean region, Pakistan, and India (46). This plant is known as “Indian Ginseng” as it is therapeutically equivalent to Ginseng (51) and was depicted as an herbal tonic for health maintenance (52). *W. somnifera* is described as an adaptogen, antioxidant, hepatic stimulant, anti-inflammatory, aphrodisiac, astringent, antifungal, and antibacterial factor (53, 54). In addition, extracts of *W. somnifera* were reported to be potent immune stimulants and anticarcinogenic (55, 56). Preparations of *W. somnifera* were also found to improve circulating antibody titer and lysosomal enzyme activity and enhance phagocytosis (57). Therefore, many studies have described *W. somnifera* extracts as immunomodulatory (58), antioxidant (59), antitumor (57), hepatoprotective (60), and antibacterial (61) agents.

Furthermore, *W. somnifera* significantly improves the blood profile in the shape of increased hemoglobin (Hb) level and increased erythrocyte and white blood cell counts (62, 63). Moreover, different parts of the herb have anti-serotonergic and anabolic characteristics and have potent impacts in the therapy of arthritis and stress, as well as geriatric problems (64). *W. somnifera* was also reported to improve circulating cortisol, lower fatigue, accelerate physical performance, and lower refractory depression in livestock exposed to various stressors (50). Similarly, *W. somnifera* is thought to strengthen the physiological and immunological functions of stressed birds (65).

## General characteristics of *W. somnifera*, active ingredients, and their activity

### *W. somnifera* morphological features and distribution

*W. somnifera* (L.) Dunal, commonly identified as “ashwagandha,” “asgandh,” or “winter cherry,” is a member of the family Solanaceae (66). It is a 30–150-cm-high, upstanding, stellate–tomentose, undershrub with long tuberous roots, opposite leaves, small greenish flowers, and orange berry-like fruits (67).

*W. somnifera* is known as a wild plant in the northwestern areas of India, expanding from the mountainous region of Punjab, Himachal Pradesh, and Jammu to an altitude of 1,500 m (68). Due to its economic and medicinal properties, it is being widely cultivated (more than 4,000 ha) in drier parts of India (69, 70).

### Chemical composition of *W. somnifera*

**Figure 1:**
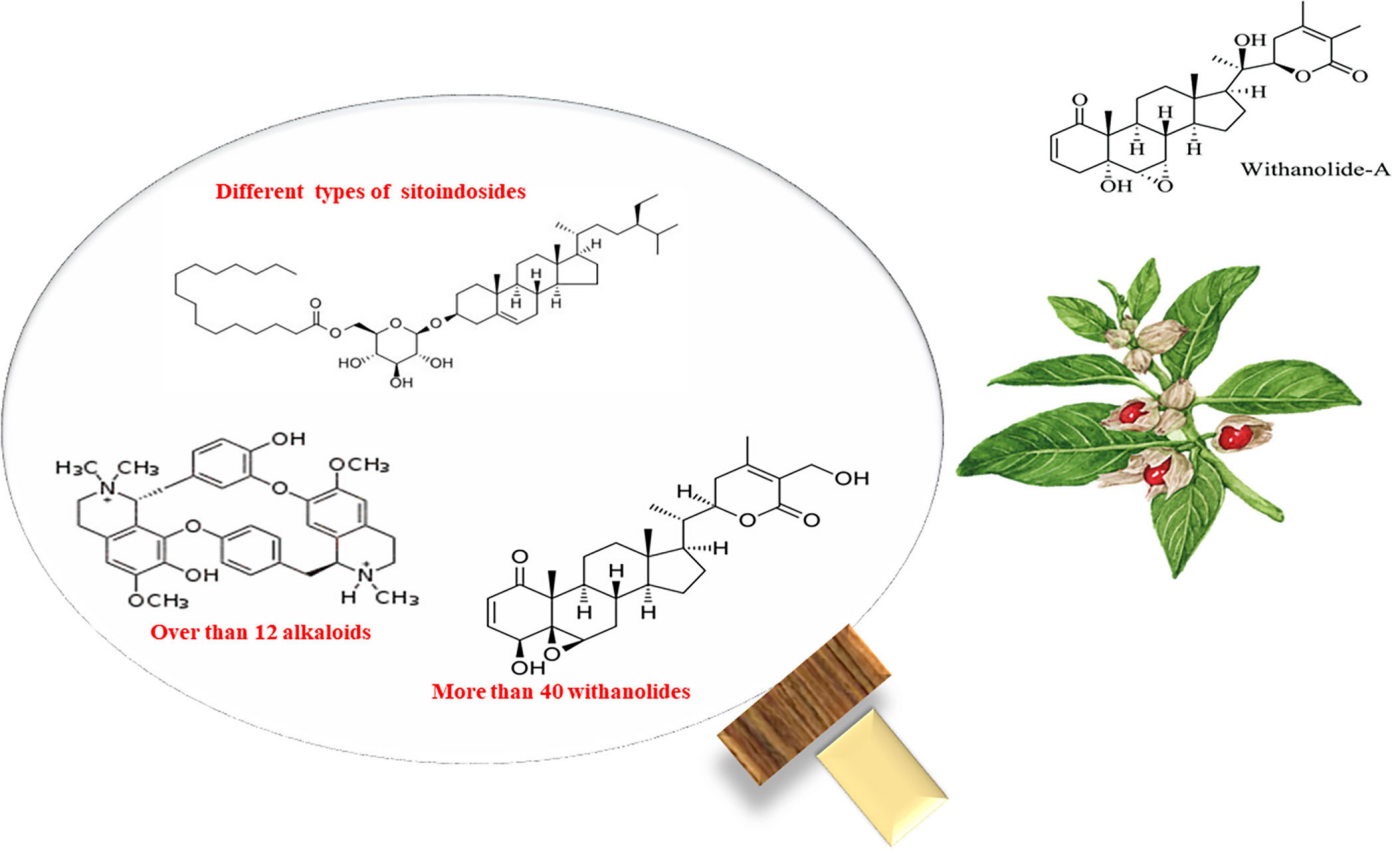
Chemical composition of *Withania somnifera*.

The chemical composition of *W. somnifera* is illustrated in Figure 1. The method of extraction of active components from *W. somnifera* plants affects the chemical composition of *W. somnifera* extracts (71). The chemical composition of *W. somnifera* has been widely investigated, and more than 39 active agents have been extracted, isolated, and identified in different studies (72, 73). Recently, different phytochemical constituents, such as total phenol, more than 12 alkaloids, 40 withanolides, and many sitoindosides, have been described (67). The withanolides are a group of naturally occurring steroidal lactones that impart a distinctive earthy odor and flavor to ashwagandha (74). These steroids comprise a lactone with a nine-carbon side chain linked to the C-17 position (71).

Different classes of withanolides have different lactone moiety variations. Withaferin A was the first member of this group to be identified (75). The Rf values of withaferin, withanolides D, and withanolides A (0.86) are 0.32, 0.50, and 0.86, respectively (76). The total alkaloid content in the roots of *W. somnifera* was found to vary between 0.13 and 0.31%, and much higher yields (up to 4.3%) were also reported (77). In addition, the *W. somnifera* roots include a small amount of soluble protein (5.6%) (76).

### Pharmacological features of *W. somnifera*

**Figure 2:**
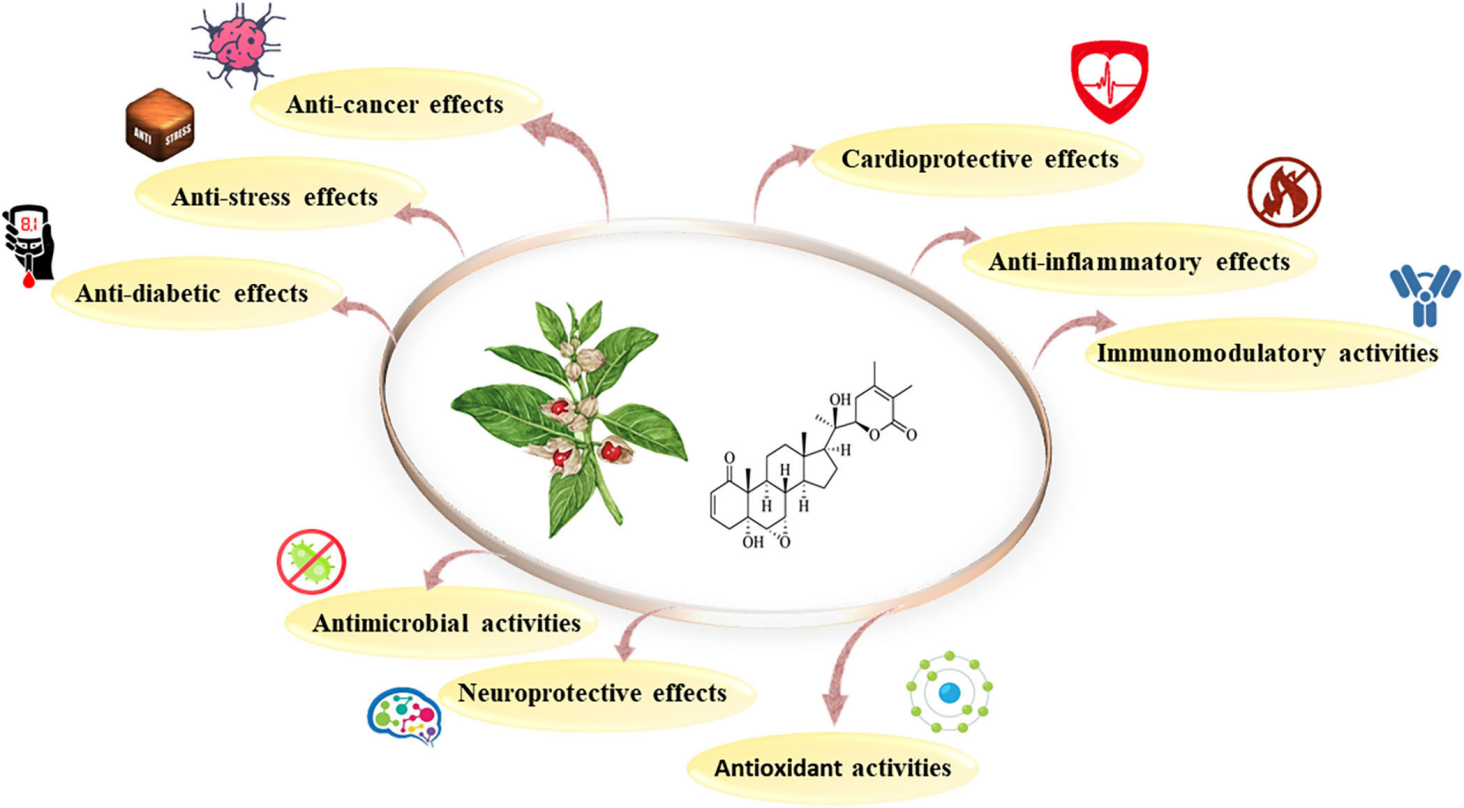
Pharmacological features of *Withania somnifera*.

The pharmaceutical features of *W. somnifera* are summarized in Figure 2. *W. somnifera* is commonly identified as a “Rasayana” in Ayurveda and is abundant in different ayurvedic products to enhance strength and stamina (52). The herb was traditionally utilized to improve youthful vigor, endurance, and strength, maintain health, accelerate the production of vital fluids, muscle, blood, lymph, and semen, and increase the capability of people to overcome environmental stress (78). The similarities between these rejuvenating features and those of ginseng roots have led to ashwagandha roots being known as “Indian Ginseng.” *W. somnifera* is also established as a general energy-stimulating tonic known as *Medhya Rasayana* that is used to promote learning and to improve memory (79).

Ashwagandha is one of the main components in 74 Ayurvedic, 9 Siddha, 3 Unani, and 126 herbal preparations (68). The roots of this plant have been regarded as a useful internal medicine in rheumatism and dyspepsia and found to be fully diuretic (80). In recognition of the importance and value of *W. somnifera* as a therapeutic agent, this plant has also been the topic of significant modern scientific interest and appeared in “WHO monographs on selected medicinal plants” (81). Recently, many pharmacological research studies recorded the cardioprotective, immunomodulatory, anti-aging, neuroprotective, and antioxidant characteristics of *W. somnifera* (78).

### Biological activities of *W. somnifera*

Several studies have described a safe, natural, and powerful antioxidant compound in ashwagandha and other plants of the family Solanaceae (78, 82) as it elevates the levels of three naturally occurring antioxidant enzymes, namely, superoxide dismutase, catalase, and glutathione peroxidase (52). In addition, oral supplementation of *W. somnifera* extract inhibited the increase in fat peroxidation in both rabbits and mice (83). The antioxidant activity of *W. somnifera* in mice was found to be imparted by glycowithanolides, withanolides, and sitoindosides VII–X (84). *Withania* usage considerably enhanced hemoglobin, red blood cell count, and hair melanin and lowered serum cholesterol level in treated individuals (85), and *Withania* root powder prohibited cadmium-stimulated oxidative stress in chickens and lead-stimulated oxidative damage in mice (86). In addition, *W. somnifera* (500 mg/kg body weight) exhibited an anti-nephron–cytotoxic effect when examined in mice with (87).

*W. somnifera* is a potent immune stimulant (78) and markedly improved the humoral-mediated (12%) and cell-mediated immune response (19.27%) (76) *via* the improvement in the numbers of neutrophil, gamma-interferon (IFN-γ), interleukin-2 (IL-2), and granulocyte–macrophage colony-stimulating factor (GM-CSF) (88). Withaferin A and withanolide D present in the root extract of *W. somnifera* increased the antimicrobial activity of immune cells by boosting nitric oxide synthase action of the macrophages (89).

*W. somnifera* is also a natural source of anti-inflammatory steroids and exhibits potent anti-inflammatory effects (90). Extracts of *W. somnifera* have an anti-inflammatory activity in different rheumatological situations (91). The extracts markedly lowered both paw swelling and bony degenerative alterations in rats with arthritis induced by Freund's adjuvant (78). Withaferin A safely and effectively suppressed the arthritic syndrome in a study on arthritic animals. Individuals treated with hydrocortisone showed weight loss, while the animals medicated with withaferin A revealed weight gain (92).

Ashwagandha was reported as a natural antidepressant and anxiolytic agent (78, 93). The root extracts of ashwagandha induce a γ-aminobutyric acid (GABA)-like activity that is responsible for the anti-anxiety effects (94). In addition, *W. somnifera* exhibits a dose-dependent antistress activity in treated mice (90). Ashwagandha roots include steroids that act as exogenous adrenocortical steroids and decrease adrenocorticotropic hormone (ACTH) secretion and, consequently, endogenous steroid production. Therefore, *W. somnifera* is considered a growth promoter, particularly during development (49).

Extracts of *W. somnifera* also presented large dose-dependent responses in different parameters such as pulse rate, blood pressure, serum cortisol, creatinine, protein, hemoglobin, and considerably higher responses in mean fasting serum lipid and blood glucose (95). Methanolic extracts of ashwagandha reduced ulcer index, volume of gastric secretion, free acidity, and total acidity in models of gastric ulcer in rats (96). Sitoindosides IX and X, two glycowithanolides from *W. somnifera*, showed a potent antistress action, caused marked mobilization and stimulation of peritoneal macrophages and phagocytosis, and improved the activity of lysosomal enzymes (97).

Withanolides have both antibacterial and antifungal activities (68). The root extract of *W. somnifera* exhibited a significant *in vitro* antibacterial activity against *Raoultella planticola, Bacillus subtilis, Enterobacter aerogens, Klebsiella pneumoniae, Agrobacterium tumefaciens*, and *Escherichia coli* (98). The minimum inhibitory concentration (MIC) of *W. somnifera* was 0.039 mg mL^−1^ against *K. pneumoniae, E. aerogens*, and *A. tumefaciens*. *W. somnifera* root extracts also demonstrated an effective antifungal activity against *Fusarium solani* (54).

*W. somnifera* root powder is traditionally used for the treatment of pulmonary tuberculosis and bubonic plague in Garhwal Himalaya (99). In broiler chicks, supplementation of 20% *W. somnifera* root extract at 20 mL L^−1^ of water lowered the severity, mortality, and recovery time of *E. coli* challenge and improved the humoral and cellular immune responses, suggesting the root extract had a protective effect in minimizing the impact of *E. coli* infection in these birds (100). *W. somnifera* also alleviated infectious bursal disease virus (IBDV)-induced stress and histological and immunological alterations and reduced IBDV persistence in the host (101). These findings were confirmed by Kumari et al. (98) in a trial with *Salmonella*-challenged broiler chickens. The *Salmonella*-challenged chickens supplemented with 0.5% *Withania* showed less reduction in the body weight (1,800 ± 130.38 g) compared with unsupplemented *Salmonella-*challenged chickens (1,600 ± 70.71 g), while a significantly higher body weight of 1,980 ± 66.33 g was observed in uninfected *Withania*-supplemented broilers compared with the control uninfected group.

*W. somnifera* alkaloids display long-standing hypotensive, bradycardic, and respiratory-stimulant activities due to the autonomic ganglion blocking effect and depressant action on higher cerebral centers (102). Ashwagandha also restored the myocardial antioxidant status and retained membrane integrity by lowering malonyl dialdehyde levels in isoprenaline-induced heart muscle necrosis in mice (103).

The glycowithanolides withaferin A (VII–X), which are found in the roots of ashwagandha, control the growth of nerve cell dendrites, exhibit a GABA mimetic effect during healing of brain tissue, and reverse neurotic atrophy or synaptic loss leading to dementia (104). Ashwagandha root extract also elevates cortical muscarinic acetylcholine receptor capacity, which leads to a cognition-enhancing and memory-enhancing activity in humans and animals (105). Ashwagandha has been considered as a tonic and nootropic agent and accompanied an enhancement in scopolamine-induced memory deficits in mice (104). *W. somnifera* methanolic extracts induce neurite extension, and dendritic atrophy could be avoided by treatment with withanolides (104).

Withaferin A also presented antitumorigenic, anticancer, and antiproliferative effects against different tumor cell lines (106) due to a depression in the expression of nuclear factor-kappa B and suppression of intercellular tumor necrosis factor, as well as potentiation of radiation-induced apoptosis in tumorous cell lines (107, 108). An alcoholic extract of *W. somnifera* had an antitumor and radio-sensitizing activity in Chinese hamster cells and Swiss mice inoculated with Ehrlich ascites carcinoma cells (109, 110). *W. somnifera* extract also reduced leucopenia induced by clophosmide in experimental animals (111).

*W. somnifera* exhibits hypoglycemic, diuretic, and hypocholesterolemic effects (112). *W. somnifera* root extracts produce hypoglycemic and hypolipidemic impacts in alloxan-induced diabetic rats (113, 114). These antidiabetic effects may be due to enhanced hepatic metabolism, improvement in insulin synthesis from pancreatic β-cells, or insulin-sparing activity (115).

## Impacts of *W. somnifera* on performance, blood parameters, and carcass quality of birds

**Figure 3:**
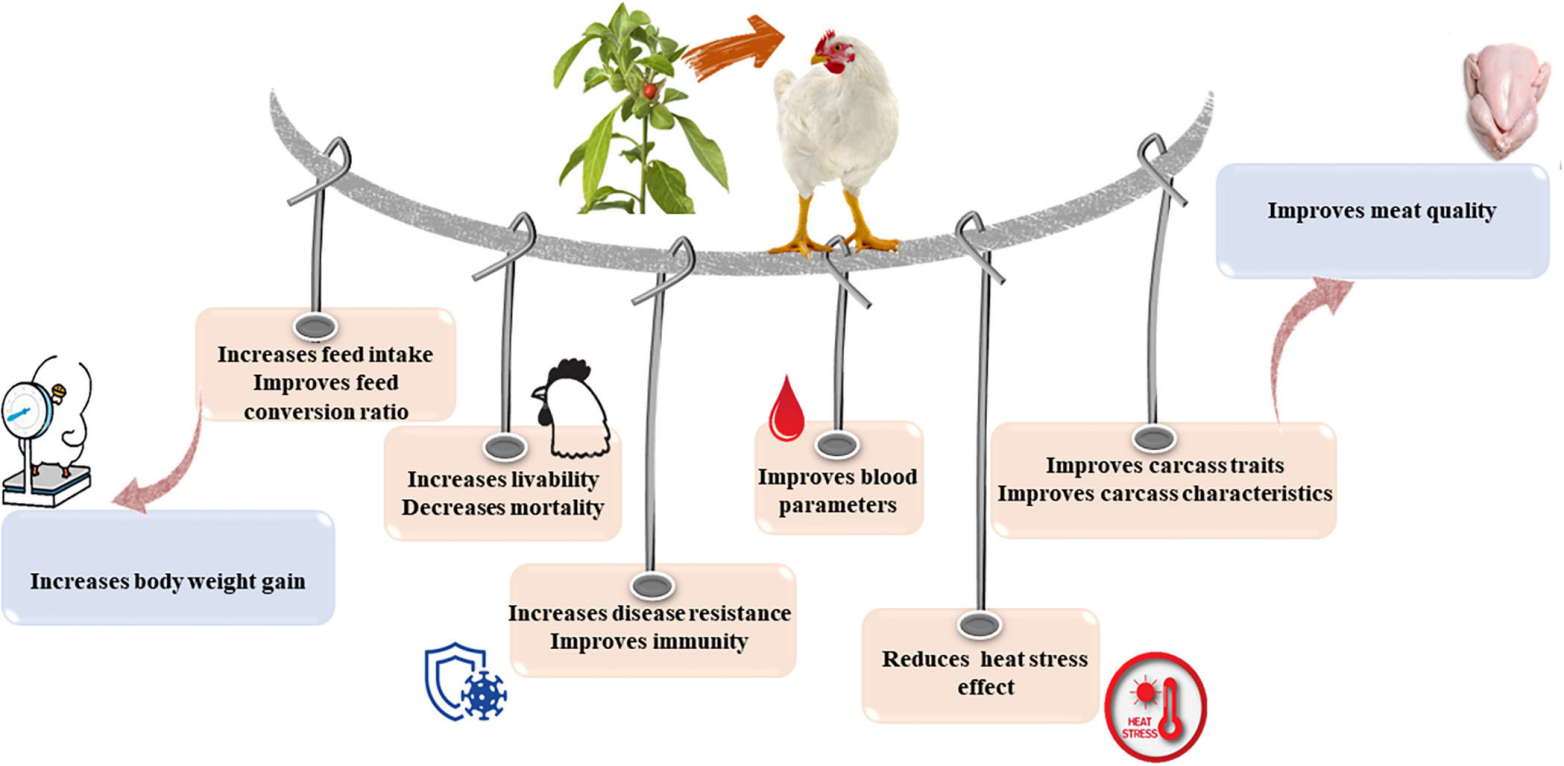
Impacts of *Withania somnifera* supplementation on birds' performance and productivity.

The impacts of *W. somnifera* on birds' performance and productivity are summarized in Figure 3 and Table 1.

**Table 1:** *Withania somnifera* effects and application.

**Type of herbal extract**	**Dose**	**Route of administration**	**Species**	**Effect**	**References**
*W. somnifera*	Extract of *W. somnifera* 20 g L^−1^ water	Drinking water	Broiler chickens	Improves feed intake and body weight	(116)
Polyherbal formulation including *Phyllanthus emblica, Ocimum sanctum, Terminalia chebula*, and *W. somnifera*	One and 2 ml/100 birds/day for 0–42 days	Polyherbal liquid dietary inclusion	Broiler chickens	Improves feed intake at the last 3 weeks of rearing period, optimizes protein and fat content of raw meat, and also maintains sensory quality of meat	(117)
*W. somnifera*	*W. somnifera-*based diet (0.5%)	Feed additives	Broiler chickens	Shows lower feed intake and improves growth parameter such as body weights and weekly gain in body weights	(118)
Medicinal plants mix including *W. somnifera, Shatavari Asparagus racemosus*, and *Mucuna pruriens*	Herbal drug (2%) along with the basal ration for 42 days	Feed additives	Broiler chickens	Improves body weight, improves the quality of meat, and fetches more economic return	(119)
*Withania somnifera, Asparagus racemosus*, and *Mucuna pruriens*	Herbal drug (2%)/kg diet/42 days	Feed additive	Broiler chickens	Shows a significant (*p* < 0.05) difference in weekly feed consumption	(120)
Ashwagandha root powder	Basal diet (0.25%– and 0.5%) for 6 weeks	Feed additives	Broiler chickens	Increases productivity, average weekly body weight gain, feed conversion ratio, and blood biochemical profile	(121)
*W. somnifera* root extract	*W. somnifera* root extract (20%) at 20 mL^−1^ of water	Drinking water	Broiler chickens	Lowers the pathogenicity, mortality, and recovery time of *Escherichia coli* challenge. Stimulates the humoral and cellular immunity and shows protective impact on lowering the pathogenicity of *Escherichia coli*	(100)
Ashwagandha and selenium	Ashwagandha (2.5%) and 0.20 mg kg^−1^ selenium in the diet	Feed additives	Broiler chickens	Improves growth performance and carcass traits	(122)
*W. somnifera*	*W. somnifera* (0, 100, and 200 mg kg^−1^) diet	Feed additives	Broiler chickens	Improves the bird productivity, immunity, and meat oxidative stability under oxidative stress status	(123)
Ashwagandha root powder	Ashwagandha root powder (0.25, 0.5, 0.75, and 1%) on feed	Feed additives	Layer chickens	Improves egg production and increase egg mass	(124)
*W. somnifera* root extract	*W. somnifera* (2.5 or 5.0%) root extract	Feed additives	Broiler chickens	Did not have any adverse impacts on productivity, immunity, serum biochemical constituents, and hematological index	(125)
*W. somnifera* root powder	*W. somnifera* (1%) of the feed	Feed additives	Broiler chickens	Alleviates infectious bursal disease virus-induced stress and histological and immunological alterations. Reduces gumboro virus persistence in the host	(126)
Shatavari and ashwagandha	Shatavari (2.5 g) + 2.5 g ashwagandha powder kg^−1^ feed	Feed additives	Broiler chickens	Improves productivity of chickens under cage conditions without having negative impact on feed conversion ratio	(127)
*W. somnifera* root powder	*W. somnifera* (0.5, 1, and 2%) root powder	Feed additives	200-day-old chicks	Enhances growth rate, feed consumption, and feed conversion and reduces mortality	(128)
Shatavari and ashwagandha	Shatavari (2.5 g) + 2.5 g ashwagandha powder/kg feed	Feed additives	Broiler chickens	Increases performance in caged conditions. Improves body weight and feed intake	(129)
Probiotic (*Lactobacillus acidophilus*) with three herbal growth promoters, Amla, Tulsi, and ashwagandha	Feed was fortified with probiotics, dried and powdered herbs as per the requirement per ton of feed	Feed additive	Broiler chicks	Herbal-treated birds revealed maximum live body weight (290 g) during second, third, and fifth weeks of the trial and improved growth performance, carcass characteristics, and dressing percentage	(130)
*W. somnifera* extract	*W. somnifera* extract (20 g)	Feed additives	Broiler chickens	Improves body weight	(131)
Ashwagandha root extract and ashwagandha root powder	Root extract (0.15%) of ashwagandha and 0.5% ashwagandha root powder	Feed additives	Broiler chickens	Improves body weight when 0.15% root extract of ashwagandha was used	(132)
*Withania somnifera* root powder (Ayucee premix)	Ayucee (100 g ton^−1^ of feed)	Polyherbal feed premix	Broiler chickens	Improves body weight and bird productivity under heat stress in the summer season	(133)
Ashwagandha root powder	Ashwagandha root powder (1%)	Feed additives	Japanese quails	Improves body weight. Improves feed efficiency. Improves the immune status	(134)
*W. somnifera* root powder	*W. somnifera* root powder (0.5%)	Feed additives	Broiler chickens under heat stress	Improves body weight, weekly body weight gain, and feed conversion ratio	(118)
*Nigella sativa, Boerhavia diffusa, W. somnifera, Ipomea digitata, Azadirachta indica*, and *Corylus avellana*	diet (4 g kg^−1^)	Feed additives	Broiler chickens	Increases production	(135)
Ashwagandha	*W. somnifera* root ethanolic extract (50, 100 mg/kg/day)	Orally	Japanese quails under heat stress	Improves body weight gain when 100 mg/kg ethanolic extract was used	(136)
*W. somnifera* leaves	*W. somnifera* leaves 0.75 g kg^−1^ leaves/kg diet	Feed additives	Broiler chickens	Increases body weight in the 4th and 5th week of age	(137)
*W. somnifera* root powder and guduchi stem powder	*W. somnifera* 1 and 2 g kg^−1^ feed	Feed additives	Broiler chickens	Improves significantly body weight. Lowers mortalities. Shows no significant differences in feed conversion ratio	(138)
*W. somnifera*	Two-fold serial dilutions of 20% aqueous *W. somnifera* root extract	*In vitro*	*In vitro* antibacterial activity testing against *Escherichia coli* O78	Shows maximum inhibition of bacterial growth at 1:8 dilution of *W. somnifera* root extract	(98)

### Impacts of *W. somnifera* on feed intake, body weight gain, and feed conversion ratio of birds

The inclusion of *Withania* in broiler feed improved feed consumption during the final 3 weeks (fourth to sixth week) of a trial (117). Sanjyal and Sapkota (130) recorded the average weekly feed consumption of 222, 432, 716, 764, and 798 g, respectively, from the second to the sixth week on a *Withania-*containing ration, with the highest digestibility (*P* < 0.05) observed in ashwagandha-supplemented chickens. FI was 7.9% higher in *Withania-*supplemented chickens compared with the control birds. Ansari et al. (135) also recorded significantly increased FI (4,580.64 g) in broilers maintained on 1% *W. somnifera* root powder-based ration compared with unsupplemented chickens (3,954.22 g).

However, Shisodiya et al. (118) recorded a reduction in FI in broilers on a 0.5% *Withania-*based diet compared with the control birds. The effect of *Withania* feeding on digestibility of the feed was also recorded by Pandey et al. (119) who observed a significantly higher body weight with concurrent significantly reduced FI (3,720.85 g bird^−1^) in broilers on a *Withania-*based diet as compared to the control birds (3,916 g bird^−1^). The average weekly FI of broiler chickens (kg/bird) from 1 to 6 weeks of age as a result of dietary inclusion of a *Withania-*based indigenous herbal drug revealed marked (*P* < 0.05) differences in the weekly feed consumption of broilers and was reported to be 0.230, 0.370, 0.530, 0.760, 0.770, and 0.960 kg, respectively, in the control birds and 0.210, 0.360, 0.510, 0.740, 0.750, and 0.930 kg, respectively, in the treated group (120). However, it was also observed that the level of *Withania* root powder supplementation at either 1 g or 2 g kg^−1^ of feed in the basal diet did not reveal a significant difference in overall FI in broiler chickens (138). The FI of Japanese quails was also improved on a 1% *Withania* root powder-containing basal diet (3,536.35 g) compared with the control group (3,154.18 g) (134). Vasanthakumar et al. (132) also recorded significantly increased FI in broilers maintained on 1% *W. somnifera* root powder-based ration compared with unsupplemented control chickens.

Ghosal et al. (97) discussed the general health tonic activity of *W. somnifera*. The results of various investigations on *W. somnifera* revealed that it has an anabolic impact and increases liver biosynthesis to raise the body weight in animals and humans (91). Moreover, numerous researchers have reported that medicinal herbs, particularly *W. somnifera*, could be employed as growth promoters in poultry diets to improve productivity. With supplementation of 0.5% *W. somnifera* root powder in broiler chicks, Shisodiya et al. (118) reported substantial improvements in growth parameters such as low birth weight (LBW) and weekly BWG. Vasanthakumar et al. (132) also referred to the beneficial effect of ashwagandha in broilers. Furthermore, Ansari et al. (135) examined the comparative efficacy of six medicinal herbs, such as *W. somnifera, Nigella sativa, Ipomea digitata, Boerhavia diffusa, Azadirachta indica*, and *Corylus avellana*, on the performance of 210-day-old broiler chickens and recorded the maximum WG in the group supplemented with *W. somnifera* (1,819 g), followed by *Nigella sativa* (1,805 g) and *Azadirachta indica* (1,800 g), when plants were supplemented at a rate of 4 g kg^−1^ of feed. Herbal drugs, including *W. somnifera, Asparagus racemosus*, and *Mucuna pruriens*, improved the body weight of VenCobb-400 broilers (120).

The synergistic effect of three different herbs, namely, ashwagandha, shatavari, and kapikachhu, on production performance of broilers was examined by Pandey et al. (119), and they concluded that ashwagandha, shatavari, and kapikachhu powder mixture in the ratio of 2:1:1 when added at a rate of 2% in the poultry ration of the VenCobb-400 broilers resulted in a higher body weight compared with that of the control chicks. The increased productivity of herbs-treated groups was attributed to the immunomodulatory, antioxidant, and antistress effects of *W. somnifera* (128, 139, 140). The feasibility of replacing antibiotic growth promoters with herbal growth promoters was discussed by Sanjyal and Sapkota (130) in a trial performed on 192 VenCobb-400 broilers with antibiotic (chlortetracycline), probiotic (*Lactobacillus acidophilus*), and three herbal (amla, tulsi, and ashwagandha) growth promoters. In this trial, the optimal live body weight (290 g) was observed in the *Withania-*treated group during the second week of the experiment and was significantly larger compared with the control and other treatments. The *Withania-*supplemented group also showed the maximum WG during the third (194 g) and fifth (412 g) weeks of the trial (130). The positive impact of *W. somnifera* on BWG of birds might be explained by the phytogenic contents of *W. somnifera* increasing the secretion of endogenous enzymes, improving hepatic function, and increasing hepatic protein biosynthesis, which are reflected in an increased BWG of the treated birds.

Rindhe et al. (117) compared the efficacy of *W. somnifera-*containing herbal formulation with synthetic ascorbic acid in a 42-day trial on VenCobb-400 broilers and found that the mean live body weight of the *Withania-*supplemented group was significantly (*P* < 0.01) higher (2,281.67 ± 4.05 g) compared with that of chickens supplemented with ascorbic acid (2,173.33 ± 4.31 g) and control birds (2,000.00 ± 8.35 g). Kumari et al. (98) recorded less reduction in the body weight (1,800 ± 130.38 g) in 0.5% *Withania-*supplemented *Salmonella-*challenged chickens compared with unsupplemented *Salmonella-*challenged chickens (1,600 ± 70.71 g) and a significantly higher body weight of 1,980 ± 66.33 g in uninfected *Withania-*supplemented broilers compared with uninfected control broilers. The body weight of broilers was significantly impacted by supplementation with 20 g *W. somnifera* extract L^−1^ water when compared with the control chicks (1,736.59 ± 0.44 g vs. 1,452.13 ± 0.89 g, respectively) (98).

Similar impacts on the body weight of broiler chicks following administration of 20 g *W. somnifera* extract were recorded by Sajjad (131) and Kakar (141). Furthermore, a 0.15% root extract of ashwagandha was significantly (*p* < 0.05) superior in improving the body weight of broilers as compared to the control and 0.5% ashwagandha root powder-fed chickens (2,297.11 ± 49.8 g vs. 1,947.83 ± 41.39 g vs. 2,214.78 ± 57.41 g, respectively) (132). These results corroborated the data of Singh et al. (85) who also recorded the elevated body weight in ashwagandha-fed chickens. A dose-dependent positive impact of *W. somnifera* on LBW and BWG in broilers was reported in several studies. A dose-related effect of *Withania* during the different weeks of a trial was reported in the study of Ahmed et al. (137). In this trial, the body weight of Ross broiler chickens in weeks 4 and 5 of the trial was affected more significantly (*P* ≤ 0.05) by the addition of *W. somnifera* to basal feed compared with the control chickens, and during the period from 3 to 4 weeks of age, chickens that received 0.75 g *W. somnifera* resulted in a significantly (*P* ≤ 0.05) higher BWG compared with control and other treated groups, whereas the final body weight and BWG at the final interval (4–5 weeks) were significantly (*P* ≤ 0.05) increased in 1.5 g *Withania-*supplemented chickens (137). The improvement in the body weight with age may be due to the impact of *W. somnifera* in stimulating the thyroid gland directly and/or through the pituitary gland to secrete more thyroid anabolic hormones (137).

Similarly, Joshi et al. (138) proved the anabolic effect of *W. somnifera* with two different doses (T2: 1 g kg^−1^ feed and T3: 2 g kg^−1^ feed) and noticed a marked (*P* < 0.05) impact on overall body weight of broilers and chicks maintained on 2 g *Withania*/kg of feed (T3), with a final body weight of 2,199.30 ± 40.20 g compared with 2,138.86 ± 34.5 g (T2) and 2,076.26 ± 22.27 g (T1: control). Average weekly BWGs were higher in *W. somnifera-*fed groups compared with the control at the first and third weeks and overall, for the trial. In addition, the total WG (g) was statistically highest (2,152.98 ± 40.27 g) in the group that received 2 g *Withania*/kg feed. On the contrary, Thange et al. (142) did not observe any impact of various doses of dietary supplementation of *W. somnifera* on the body weight in broilers. The dietary supplementation of ashwagandha not only improved the body weight in the thermo-comfort zone but also accelerated the body weight in temperature extremes. Furthermore, a polyherbal premix containing *W. somnifera* root powder added to the chicken feed significantly improved the body weight of broilers after a 6-week trial in the summer when the mean temperature–humidity index (84.74 ± 2.51) was greater than the thermo-comfort zone of broilers (133).

Japanese quails also exhibited an improvement in performance with the supplementation of ashwagandha. The addition of 1% ashwagandha root powder significantly (*P* < 0.05) enhanced the body weight of Japanese quail chicks (134). Similarly, Ahmed et al. (136) reported a significant (*P* ≤ 0.05) improvement in BWG of quails fed with a 100 mg kg^−1^ ethanolic extract of ashwagandha as compared to the control birds.

The FCR (amount of feed intake/unit LWG) ultimately determines the economics of the broiler industry. A significant reduction in FCR was recorded by Shisodiya et al. (118) in broiler chicks when the basal diet was supplemented with 0.5% *Withania* root powder. Comparison of the effect of *Withania* and five different herbs, such as *Corylus avellana, Boerhavia diffusa, Ipomea digitata, Azadirachta indica*, and *Nigella sativa*, in broilers also revealed a significantly better FCR during most weeks in the birds fed the *Withania-*included diet (135). Rindhe et al. (117) reported a lower FCR (2.05) in ashwagandha-fed birds compared with ascorbic acid-supplemented and control broilers. A comparative study conducted by Sanjyal and Sapkota (130) in broiler chickens resulted in an improved FCR in a group fed with *Withania* root powder compared with antibiotic and two other herbs, namely, amla and tulsi.

Srivastava et al. (120) recorded enhanced weekly FCR from the first to the sixth week of age in *Withania-*treated broilers. The overall FCR (1.74) during all the weeks was statistically very low in broilers reared on 2% herbal formulation containing 50% *Withania* powder compared with the control birds (2.07) (119). A numerically improved feed conversion efficiency was recorded by Vasanthakumar et al. (132) in broiler chickens reared on 0.15% ashwagandha root extract. However, the graded level of *Withania* supplementation at 1 g kg^−1^ of feed and 2 g kg^−1^ of feed did not result in a significant (*P* > 0.05) difference in FCR in broiler chickens (138). Non-significant differences in FCR were also reported by Thange et al. (142). The improvement in feed efficiency was also observed in Japanese quails (134) with the addition of *W. somnifera*. 0.5%, 1.0%, and 1.5% *Withania* root powder feed supplementation (143). The enhanced FCR (*P* ≤ 0.05) was also reported when quails were supplemented with the ashwagandha root ethanolic extract (100 mg and 200 mg kg^−1^ feed) or with 2 g kg^−1^ diet of root powder in contrast to controls (136). A significant increase in FI of 3,231.27 ± 0.44 g was recorded in broiler chicks supplemented with a 20 g extract of *W. somnifera* L^−1^ water when compared with the control group (2,864.91 ± 0.89 g) (144).

To summarize, the overall improvement in the performance of poultry supplemented with dietary *W. somnifera* could be due to the impact of *W. somnifera* on increasing the level of the anabolic hormones, enhancing endogenous enzyme production, increasing nutrient digestion and absorption, improving liver function, increasing antioxidant capacity, and elevating hepatic protein biosynthesis.

### Impacts of *W. somnifera* on hematological and biochemical blood parameters

The hematinic activity of *W. somnifera* on broiler chickens was recorded by Kumari et al. (98), who found a significantly higher Hb level, the packed cell volume (PCV), and non-significant mean corpuscular volume (MCV) and mean corpuscular hemoglobin concentration (MCHC) values between control and *Withania-*treated birds. The hematinic activity of *W. somnifera* root powder is attributed to direct and indirect action on the hematological parameters. A direct positive impact of *W. somnifera* was noticed on hemopoiesis in broiler chicks *via* stimulation of stem cell proliferation and improved bone marrow cellularity (49, 144). Also, *W. somnifera* root powder protected red blood cells from oxidative stress in broiler chickens through its antioxidant effect and improvement in the erythrocytic enzyme activity (133). Daisy (145) in broilers and Bhardwaj et al. (134) in Japanese quails reported significant improvements in total erythrocytic numbers. Less intense anemia was recorded in *Salmonella-*infected broiler chickens raised on ashwagandha root powder, with the chicks rapidly recovering from *Salmonella* infection (98). In broilers treated with the extract of *Withania* root powder (10, 20, and 30 g L^−1^), there was no significant difference in Hb levels (116). In contrast, Bhardwaj et al. (134) discovered a considerable increase in Hb content in Japanese quails. The PCV value of broilers treated with *Withania* extract at 10 and 20 g L^−1^ was considerably greater compared with that of the equivalent control group chicks (116).

Bhardwaj et al. (134) found a significant and linear rise in PCV in Japanese quails after adding increasing amounts of ashwagandha root powder (0.5, 1.0, and 1.5%) compared with the untreated quails. Marked elevations in phagocytic cell counts (55, 146, 147), along with an increase in phagocytic potential, were reported in avian species supplemented with *W. somnifera* (128). Furthermore, Gautam et al. (62) recorded a marked elevation in numbers of white blood cells of broilers. A higher mean total leukocyte count in chicks supplemented with 20 g L^−1^ *Withania* root extract was recorded; however, differences in the levels of monocytes, neutrophils, eosinophils, and lymphocytes in *Withania-*supplemented chicks were not significant when compared with the values found in the control chicks (116). The level of lymphocytes in broilers treated with 1.5% ashwagandha was significantly elevated up to 53.59% with no change in heterophil and monocyte levels (134).

The considerable hypoglycemic effect (12%) of *W. somnifera* root powder observed in human subjects was infrequently confirmed in broilers (112). The blood glucose level in broilers at the end of the sixth week of a trial was unaffected by a herbal preparation including *W. somnifera* root powder supplemented at 2% in basal diet (120). A similar non-significant role of ashwagandha on serum glucose levels was recorded in guinea pigs (148). Furthermore, broilers treated with ashwagandha leaves also showed non-significant alterations in blood glucose levels (137). However, lower plasma glucose (182.18 mg dl^−1^) was reported in broiler chickens treated with *Withania* at 0.01% of diet compared with that of control birds (249.52 mg dl^−1^) (133). The hypoglycemic impact of ashwagandha in broilers was predominantly reported under stress (149).

The elevation in serum protein following administration of *Withania* is due to the direct anabolic effect of ashwagandha or occurs indirectly through an increase in thyroid hormone level (150). During experimental hyperglycemia, *W. somnifera* root extract was reported to effectively reverse increased proteolysis and lower protein levels and improve serum albumin and total protein levels, which never strayed from the normal range during the experiment (113). The serum protein regulatory activity of ashwagandha was confirmed by Verma and Gaur (76) in pesticides-intoxicated cockerels, with 20 mg *Withania* root extract/bird/day producing a marked elevation in serum protein levels in the cockerels. In *Salmonella*-infected broilers, 0.5% ashwagandha root powder had a strong resistive effect on serum protein and albumin levels, as well as a marked elevation in serum globulin level (98). However, ashwagandha leaves did not confer this protein-modulating role (137). Significant rises in serum total protein and globulin concentrations with numerical elevation in albumin level were observed in broilers raised on *W. somnifera* root powder (151), and *W. somnifera* root extract at 20 mg/day/bird for month significantly accelerated serum total protein to 24.42 g/100 mL compared with 15.7 g 100 mL^−1^ in control cockerels (150).

The anabolic impact of ashwagandha was more effective under stress in broilers. In addition, a significant recovery from enrofloxacin-induced hypoproteinemia was reported in broilers treated with ashwagandha (152). Reductions in the severity of depression in serum total protein and albumin were recorded in *Salmonella gallinarum-*challenged broilers on ashwagandha supplementation (98). *Withania-*supplemented broiler chickens revealed higher plasma protein and total globulin levels compared with the control birds (133). Locally prepared herbal drugs, including *W. somnifera, Mucuna pruriens*, and *Asparagus racemosus*, supplemented at 2% of broiler ration resulted in non-significant differences in serum total protein among control and treated birds (120).

The total plasma cholesterol at 0.01% ashwagandha of broiler ration was significantly decreased compared with that of untreated control birds (133). Moreover, 2% *W. somnifera* root powder supplementation in layers revealed a 30% reduction in egg cholesterol concentrations and 26% lowering in egg-yolk triglycerides (153). Research in humans and rats verified the hypocholesterolemia and hypolipidemic impact of ashwagandha root powder (112, 113). The addition of 0.5% ashwagandha root powder markedly lowered the concentration of two major negative hepatic health indicator enzymes, namely, serum alanine aminotransferase (ALT) and aspartate aminotransferase (AST), in broilers infected with *Salmonella gallinarum*, while lactate dehydrogenase (LDH) activity remained markedly higher until the end of the experiment (35 days) and a significantly low decline in alkaline phosphatase (ALP) was recorded (98).

The hepatoprotective and cardioprotective activity of ashwagandha is due to the presence of alkaloids, withanolides, and free-radical scavenging characteristics of ashwagandha (60). *E. coli-*challenged guinea pigs and treated with *W. somnifera* also revealed a similar decrease in ALT and AST concentrations (148). Supplementation of ashwagandha in pesticides-intoxicated cockerels markedly reduced the toxic impact of the pesticides in terms of lowering ALT and AST concentrations with a concurrent significant appreciation in the activity of ALP related to development (149). The ALT- and AST-reducing effect of roots of *W. somnifera* was not observed with leaves of ashwagandha in broiler chickens (132). In contrast, a trial on a herbal preparation containing *W. somnifera* did not significantly impact serum ALT and AST in broiler chickens fed at 2% per kg of ration (120). A calcium-sparing impact of ashwagandha was recorded by Varma et al. (149).

Finally, the positive impact of *W. somnifera* on hematological and biochemical blood parameters could be attributed to the hematinic activity of *W. somnifera* in stimulating stem cell proliferation, improving bone marrow cellularity, elevating antioxidant capacity that delays lipid oxidation, increasing erythrocytic enzyme activity, improving phagocytic activity, elevating white blood cells production, regulating serum proteins, and reducing total plasma cholesterol and its different alkaloids. In addition, the withanolide contents of *W. somnifera* act as free-radical scavengers that mitigate the oxidative stress impacts and show hepatoprotective and cardioprotective effects.

### *W. somnifera* antioxidant potential and its impacts on carcass characteristics and meat quality

Following exposure to acute and chronic heat stress, significant negative impacts on livability, productivity, immunity, and illness susceptibility were observed in poultry (154). Heat stress might contribute to the inferiority of acquired immunity in high-meat-yielding broiler lines. Heat stress lowered both cell-mediated and humoral immunity in birds, explored through evaluation of phagocytic activities and serum antibody titers, respectively (155). Significant amelioration (*P* < 0.05) in recovery from *Salmonella gallinarum* experimental infection was observed at 28 days post-infection of broilers supplemented with ashwagandha root powder (156).

The usage of different antioxidants in Cobb male broilers revealed a linear increase in serum T3 and T4 under heat stress (157). Furthermore, the use of a herbal preparation at 0.01% in basal feed—containing *W. somnifera* as *one* of the main ingredients—under thermal stress (84.74 ± 2.51 temperature–humidity index) significantly accelerated serum total protein and serum globulin in broiler chickens compared with the control birds, while there was non-significant variation in albumin content between treated and control broilers (133).

Ashwagandha protects broilers in terms of lowering mortality due to infection-related stress and promotes early recovery from disease. A ten-fold lower mortality (1.42%), relative to the control (14.28%), was reported by Pandey et al. (119) in broilers supplemented with ashwagandha. Kumari et al. (98) observed a considerable decline (50%) in mortalities of broiler chickens when the birds were supplemented with 0.5% *W. somnifera* root powder. The antistress and adaptogenic effect of ashwagandha lowered the severity of the infection and facilitated the early recovery of broilers from experimental infection with *Salmonella gallinarum*. Also, the cumulative mortalities in broilers were reported to be 4.4, 2.2, and 2.2% in control, 0.1%, and 0.2% ashwagandha-fed broilers, respectively (138). Similar results were recorded in mice treated with *W. somnifera* during experimental salmonellosis, indicating that supplementation with *W. somnifera* might have a promising impact in various species (61, 120).

Sanjyal and Sapkota (130) reported a higher dressing percentage in *Withania-*raised broilers (78%) as compared to the control birds (76%). A similar finding was reported by Ahmed et al. (137) who showed a non-significant elevation in dressing percentage in birds supplemented with 1.5 g ashwagandha leaves (76.41%) when compared with the control birds (75.23%). The leg weight of control (23.46%) and ashwagandha-fed broilers (22.20%) was also not significantly different (130). Congruent with this, non-significant differences in breast (40.18 and 37.04%) and thighs cut percent (25.90 and 27.60%) were reported in treated and control broilers (137). Conversely, Rindhe et al. (117) recorded a positive impact of a polyherbal antistress and antioxidant preparation containing *W. somnifera, Ocimum sanctum, Terminalia chebula*, and *Phyllanthus emblica* in increasing the carcass yield, dressing percentage, and filet, tender, and giblet yields. In the supplemented group, carcass yield was improved by 29.64%, dressing percentage by 0.83%, filet yield by 23.2%, tender yield by 12.88%, and giblet yield by 10.8%.

Similar findings of higher dressing percentage, breast weight, and leg weight were reported in groups fed with 10 ml plant extract (62.3%) when compared with control (51.11%) (158). The weight of liver in 1% ashwagandha and 0.15% ashwagandha extract (2.50)-supplemented broiler groups showed non-significant increases in the examined measures compared with the control chickens (132). Also, Sanjyal and Sapkota (130) found statistically similar percentage relative weights of liver, heart, and gizzard in broilers raised with *W. somnifera*. Vasanthakumar et al. (132) recorded a non-significant alteration in intestinal length of carcasses of broilers supplemented with ashwagandha as compared to the control birds; the observed intestinal lengths were 183.75, 213.50, and 221.33 cm in birds fed with control, ashwagandha root powder at 1% of feed, and ashwagandha root extract at 0.15% of feed, respectively.

**Figure 4:**
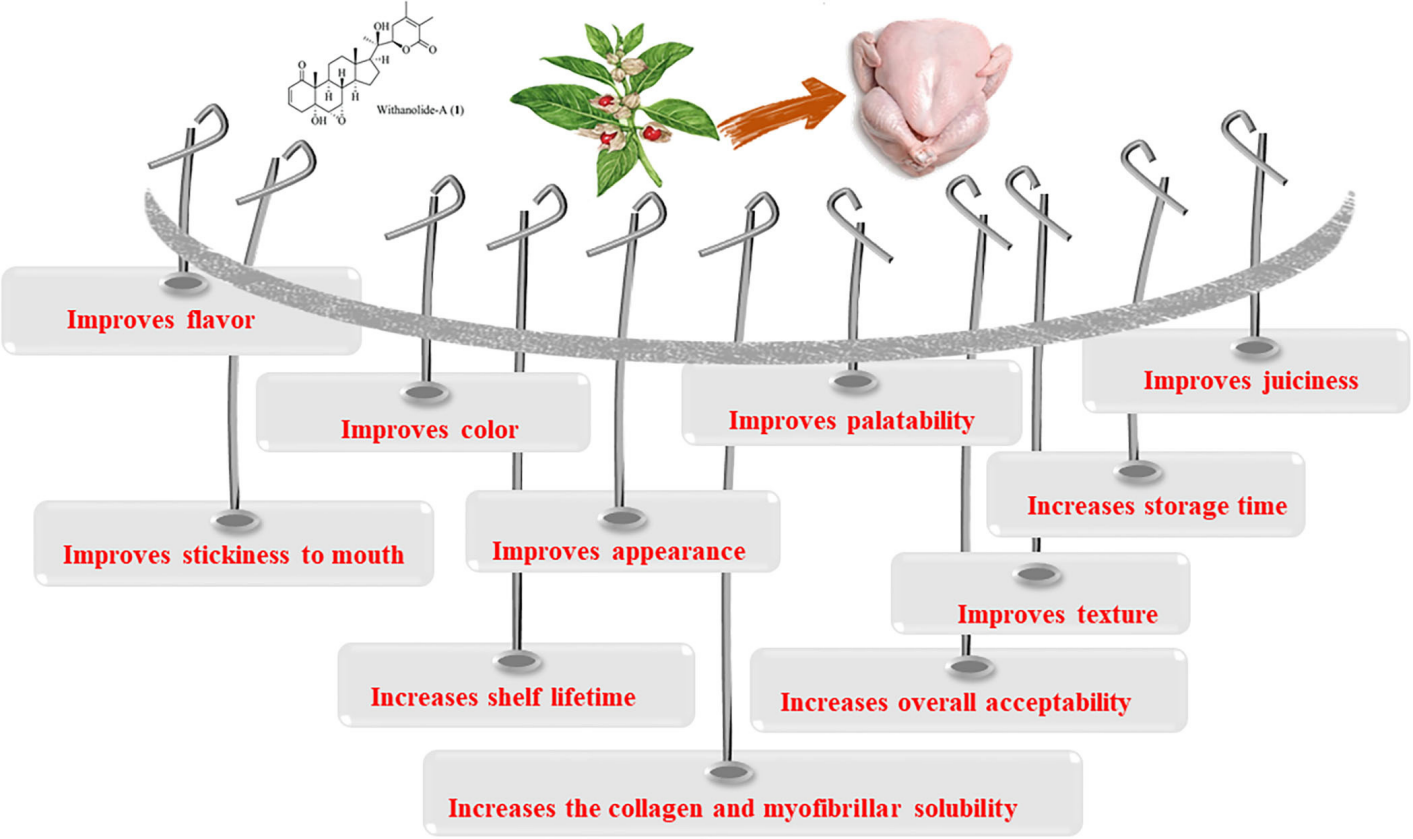
Impacts of *Withania somnifera* supplementation on broiler meat quality.

The impact of *W. somnifera* on broiler meat quality is represented in Figure 4. The addition of ashwagandha to the basal feed of broilers significantly affects the sensory qualities of broiler meat. Meat from the broilers fed herbal feed additive containing *W. somnifera* was reported to be superior to the control with respect to all attributes, including flavor (6.72 and 5.90 for supplemented and control groups, respectively), appearance (7.32 and 6.5), tenderness (7.13 and 6.14), juiciness (7.30 and 7.01), stickiness to mouth (7.24 and 6.11), and overall acceptability (7.5 and 6.03) (119).

Another sensory evaluation of broiler meat revealed significant increases in organoleptic traits of broiler meat, i.e., appearance (6.10 and 6.48 for control and treated groups, respectively), odor (5.8 and 6.81), color (6 and 6.81), flavor (5.66 and 6.5), juiciness (6.1 and 6.83), texture (6 and 6.8), and overall palatability (6 and 6.6), in groups treated with plant products AV/LAP/19 including ashwagandha, compared with the control group (117). Improved tenderness with palatability was attributed to increases in collagen and myofibrillar solubility of meat due to AV/LAP/19 supplementation (117). The oxidative stability of broiler meat expressed in terms of thiobarbiturate acid (TBA) level displayed significantly lower values in the AV/LAP/19-treated group at the end of the 15th, 30th, 45th, and 60th storage days (0.33, 0.35, 0.42, and 0.54 mg malonaldehyde/kg, respectively) in comparison with those of the control group (0.31, 0.39, 0.50, 0.60, and 0.66 mg malonaldehyde/kg, respectively) (117).

In addition, a reduced level of tyrosine in broiler meat, which is indicative of less proteolysis, was recorded upon supplementation of AV/LAP/19 plant product. Thus, the reduced TBA and tyrosine level of broiler meat reported in the AV/LAP/19-treated group was found to improve the shelf life of frozen raw meat (117). Inclusion of the *W. somnifera* at 100 or 200 mg/kg in the diet of the broilers negated the negative impacts of oxidized oil by reducing the MDA content in thigh meat and increasing the activity of antioxidant enzymes, thereby improving the performance, immune reaction, and meat oxidative stability of broilers exposed to oxidative stress (53, 159).

### *W. somnifera* immune modulation features

Manoharan et al. (53) reported an elevated antibody titer following consumption of *W. somnifera* extract in various avian models. *W. somnifera* extract at 10, 20, and 30 g L^−1^ effectively improved the antibody titer against infectious bursal disease (IBD) (116). The immunoglobulin concentrations were higher in 1.5% ashwagandha-fed Japanese quail compared with the control birds (134). The immune status of broilers as expressed by antibody titer values (log_2_) was enhanced in 1% ashwagandha root powder-treated (7.3) and 0.15% ashwagandha extract-treated (7.0) groups as compared to the control birds (6.6) (132). In addition, 1% ashwagandha root powder-raised broilers exhibited better immunity compared with the control birds (139), and humoral immunity of broilers was improved with ashwagandha root powder supplementation (159). In broiler during summer stress, total immunoglobulin was elevated following 0.01% *W. somnifera* supplementation (3.83) as compared to the control (2.79) (133). Okonkwo et al. (160) concluded that a high antibody titer could be obtained in broiler groups raised on herbal preparations including ashwagandha.

### Impact of *W. somnifera* on broiler economics

**Figure 5:**
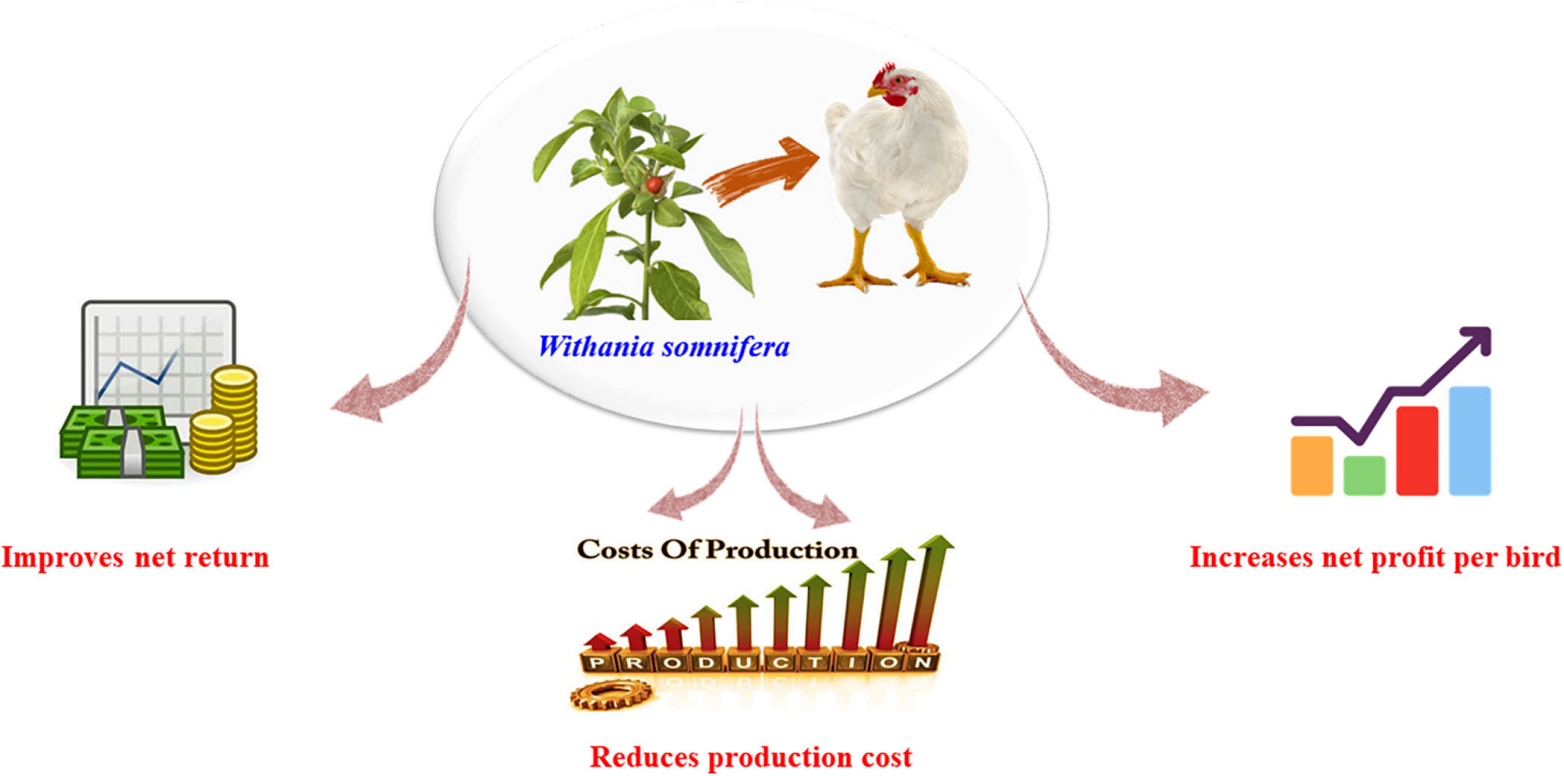
Impacts of *Withania somnifera* supplementation on economic efficiency of broilers.

The impact of *W. somnifera* addition on economic efficiency of broilers is expressed in Figure 5.

Pedhavi et al. (161) reported a better net return upon treatment with a 20% root extract of *W. somnifera* in broilers. The improved net return was also reported by Javed et al. (158) following combined treatment with *W. somnifera* and *Berberis lycium* compared with their individual outcomes, which could be attributed to efficient feed utilization by the broiler chickens at 10% extract of the tested herbs. Ansari et al. (135) performed economic evaluation and showed a maximum profit per bird in *W. somnifera* root powder-raised broiler chickens (Rs. 21.44) compared with broilers fed with *Nigella sativa* (Rs. 20.60), *Azadirachta indica* (Rs. 20.38), or control birds.

In another study, net return was highest in the ashwagandha-treated group (Rs. 48.48), followed by synthetic growth promoters (Rs. 47.92) and then control birds (Rs. 47.34) (118). Mane et al. (162) also noted a higher net profit per bird in broilers fed with ashwagandha. In contrast, Kale et al. (163) reported less net profit per bird fed with ashwagandha (Rs. 15.60) compared with the control (Rs. 16.55); however, gross return was significantly higher in 0.25% ashwagandha-treated broiler chickens (Rs. 110.10) compared with the control group (Rs. 107.58). A higher cost of production observed for probiotic-supplemented (Rs. 141.8) and ashwagandha-supplemented (Rs. 134.7) groups as compared to the control group (Rs. 128.3) was due to the extra cost incurred on the usage of ashwagandha root powder and probiotics (130).

## Conclusion

*Withania somnifera* is rich in valuable active components such as alkaloids and withanolides that act as free-radical scavengers, increase antioxidant capacity, stimulate the secretion of endogenous digestive enzymes, increase nutrient digestibility, improve blood parameters, enhance immunity, mitigate the negative impacts of stress, and alleviate the impact of diseases. Therefore, incorporation of *W. somnifera*, especially at a level of 2.5 g kg^−1^ feed in poultry ration or 20 ml l^−1^ in the drinking water, improves the livability, productivity, carcass traits, meat quality, disease resistance, blood parameters, and immunological status of the treated bird. Further, investigations should be adopted to determine the mechanism of action of potential active components of *W. somnifera* extracts and suggest the ideal dose and application method of these ingredients to obtain maximum beneficial effects.

## Author contributions

All authors contributed equally to this review and have read and agreed to the published version of the manuscript.

## Conflict of interest

The authors declare that the research was conducted in the absence of any commercial or financial relationships that could be construed as a potential conflict of interest.

## Publisher's note

All claims expressed in this article are solely those of the authors and do not necessarily represent those of their affiliated organizations, or those of the publisher, the editors and the reviewers. Any product that may be evaluated in this article, or claim that may be made by its manufacturer, is not guaranteed or endorsed by the publisher.
